# Severe degranulation of mesenteric mast cells in an experimental rat mammary tumor model

**DOI:** 10.55730/1300-0144.5921

**Published:** 2024-09-10

**Authors:** Senem Esin YAVAŞ, Özkan YAVAŞ, Semiha ERSOY, Gürsel SÖNMEZ

**Affiliations:** 1 Department of Histology and Embryology, Faculty of Medicine, Bursa Uludağ University, Bursa, Turkiye; 2 Department of Pathology, Faculty of Veterinary Medicine, Bursa Uludağ University, Bursa, Turkiye

**Keywords:** Mast cell, mast cell activation syndrome (MCAS), 7, 12-DMBA, mammary tumor

## Abstract

**Background/aim:**

Breast cancers are one of the most common cancers in women and are responsible for many deaths worldwide. Mast cells are inflammatory cells. Their role in cancers is controversial, and there is limited data on systemic mast cell activation in cancer cases. This study aimed to evaluate systemic mast cell activation in an experimentally induced rat model of breast cancer.

**Materials and methods:**

Sprague Dawley female rats were divided into control (n = 6) and mammary tumor (n = 12) groups. In the tumor group, 20 mg 7,12-dimethylbenz[a]anthracene (DMBA) dissolved in 1 mL cottonseed oil was administered intragastrically by gavage, and the rats were followed daily until their mammary tumors reached 3 cm in diameter. The control group received only cottonseed oil. Paraffin sections obtained from the mammary tumor tissue were subjected to hematoxylin-eosin, toluidine blue staining, and proliferating cell nuclear antigen (PCNA) immunohistochemistry. Mesenteric tissues from each subject were also stained with toluidine blue. The number and activation status of mast cells in mammary tumors and mesenteric tissues were evaluated.

**Results:**

Toluidine blue staining showed that activated mast cells were commonly found in tumor tissues. Based on the mesenteric tissue analysis, severe degranulation of the mesenteric mast cells was found in the tumor-induced groups compared to the control group.

**Conclusion:**

This study demonstrated for the first time that systemic mast cell activation develops in both tumoral and mesenteric tissues in an experimental cancer model. However, it is not known at which stage of tumor development it occurs.

## 1. Introduction

Breast cancer is one of the most common causes of morbidity and mortality in women worldwide. It caused more than 600,000 deaths worldwide in 2022 and more than 300,000 deaths in the United States in 2024 [1,2]. It is a heterogeneous disease with many subtypes. The aggressiveness of cancer is determined by tumor cell-specific characteristics and the complex crosstalk of cancer cells with the microenvironment consisting of the extracellular matrix (ECM), migrating cells, and the soluble factors released from them. Since tumors have evolved multiple mechanisms to grow and spread by evading the immune system, there is a strong interest in research on the interaction between cancer and immune system cells [3,4]. The chemical 7,12-dimethylbenz[a]anthracene (DMBA) is a carcinogen that acts via the DNA acylation mechanism and is widely used for the induction of mammary tumor development in female rats [5]. Since DMBA-induced mammary tumors in rats do not show excessive invasion from mammary adipose tissue, rarely metastasize, and are highly hormone-dependent, they are used in the modeling of estrogen-dependent human breasts [6,7].

Mast cells are granulocytic effector cells known for their essential contribution to IgE-dependent anaphylactic reactions, infections, asthma, and various allergies [8]. Recent studies have shown that mast cells infiltrate tumors and tumor microenvironments and release many mediators (e.g., EGF, NGF, PDGF, SCF, angiopoietin, heparin, IL-8, and VEGF), thereby affecting tumor development, tissue remodeling, and tumor-adaptive immune responses [9,10].

Mast cell activation syndrome (MCAS) is characterized by the abnormal release of variable subsets of mast cell mediators in combination with morphologically altered and immunohistochemically identifiable mutated mast cells due to mast cell proliferation (systemic mastocytosis) [11]. Despite numerous studies reporting the invasion of mast cells into intratumoral and peritumoral areas in various cancers and their possible anti- and pro-tumorigenic effects, data on the systemic status of mast cells in cancers are limited [12–14]. Recent studies supported the idea that mast cells are involved in tumorigenesis, based on the knowledge that patients with MCAS have an increased cancer risk [11]. Other study showed that increased systemic mastocytosis is associated with an increased risk of solid cancer [15]. However, there are no studies directly linking this activation syndrome with cancer formation. Therefore, the aim of this study was to evaluate the activation of systemic mast cells in a 7,12-DMBA-induced rat mammary cancer model. For this purpose, histopathologic analysis of mesenteric tissue, where mature mast cells are abundant, was deemed appropriate.

## 2. Materials and methods

### 2.1. Experimental group

Eighteen female Sprague Dawley rats aged six weeks were used in the study. The number of experimental animals to include in each group was based on similar studies [7,16]. In addition, the number of animals required for the groups was roughly calculated by the resource equation method [17,18]. The animals were randomly divided into control (n = 6) and mammary tumor (n = 12) groups. The rats were housed under standard laboratory conditions and provided unlimited access to food and water during all stages of the experiments. The research was approved by the Animal Experiments Local Ethics Committee of Bursa Uludağ University on February 25, 2020, with the approval number 2020-04/03.

### 2.2. Experimental study

Mammary tumor induction was performed by intragastric administration of 20 mg DMBA dissolved in 1 mL cottonseed oil via gavage to the 12 rats that would constitute the tumor group. The tumor diameters were measured daily with a clipper and the tumors were excised when they reached 3 cm diameter. Only cottonseed oil was given to six rats, who were designated as the control group.

### 2.3. Collection of mammary tumor tissue

The rats were euthanized when the tumor diameter was 3 cm, and mammary tumor tissues were excised. After two days of fixation in 4% paraformaldehyde at 4 °C, the tissues were routinely processed and blocked in paraffin.

### 2.4. Histopathological and immunohistopathological analyses

Paraffin mammary tumor sections of 5 μm in thickness were stained with hematoxylin-eosin to evaluate general pathological findings and Toluidine blue to identify mast cells in the tumor tissue.

Proliferating cell nuclear antigen (PCNA) immunohistochemistry was performed to demonstrate the proliferation of neoplastic cells. For the recovery of antigenicity in the immunohistochemistry analyses, a mouse anti-PCNA primary antibody (Santacruz Biotech, Dallas, TX, USA) was incubated overnight at 4 °C at a dilution of 1:300 in a pH 6.0 citrate buffer after 5 min of microwave irradiation to quench endogenous peroxidase and protein blocking. After applying HRP polymer (Thermo Ultravision, Abington, UK), two researchers evaluated the sections visualized with 3,3’-diaminobenzidine (DAB) using a photomicroscope (Olympus Corporation, Tokyo, Japan). Working from randomly selected areas of each tumor tissue section, 1000 cells were counted, and PCNA-positive cells were expressed as a percentage of the total number of cells.

### 2.5. Mesenteric tissue acquisition and mast cell staining

Mesentery and intestinal tissues were removed from approximately the same area of all subjects in both groups, washed in saline and placed in Petri dishes containing 4% paraformaldehyde for 2 h. For staining, the tissues were washed with distilled water and kept in a pH 2.5 toluidine blue solution for 5 mins. After decolorization in distilled water followed by decolorization in alcohols, the mesentery tissues were carefully separated from the intestinal tissues, spread on slides, dried, and covered. The number and activation status of mast cells were evaluated in the perilymphatic and mesenteric tissues.

### 2.6. Determination of the severity of mesenteric mast cell degranulation

Scoring to grade the level of mast cell degranulation was performed as previously described [19]. The mesenteric tissue samples were evaluated by light microscopy, and the mast cells were scored at 20× and 40× magnifications. The severity of mast cell degranulation was numerically scaled from 0 to 4. Mast cells with intact granulated morphology were rated as 0; very mildly degranulated mast cells were rated as 1; mostly intact mast cells with mild degranulation were rated as 2; moderately degranulated mast cells were rated as 3; and mast cells with severe degranulation were rated as 4. Histological analysis was performed by two independent observers.

### 2.7. Statistical analysis

To determine whether the results obtained were significant, the mean ± standard deviation of the data was calculated and the statistical difference between the groups was calculated using the SPSS 23.0 program (IBM, Armonk, NY, USA). First, the Shapiro–Wilk Test was used to determine whether the data belonging to the groups were normally distributed. Then, the statistical differences between the groups were measured with the Mann–Whitney U test. Statistical significance was set at p < 0.05.

The relationship between two variables was evaluated with correlation analysis. First, after determining whether the data showed normal distribution, a Pearson correlation analysis was applied. Statistically significant difference between two variables was set at p < 0.05.

## 3. Results

**Figure 1:**
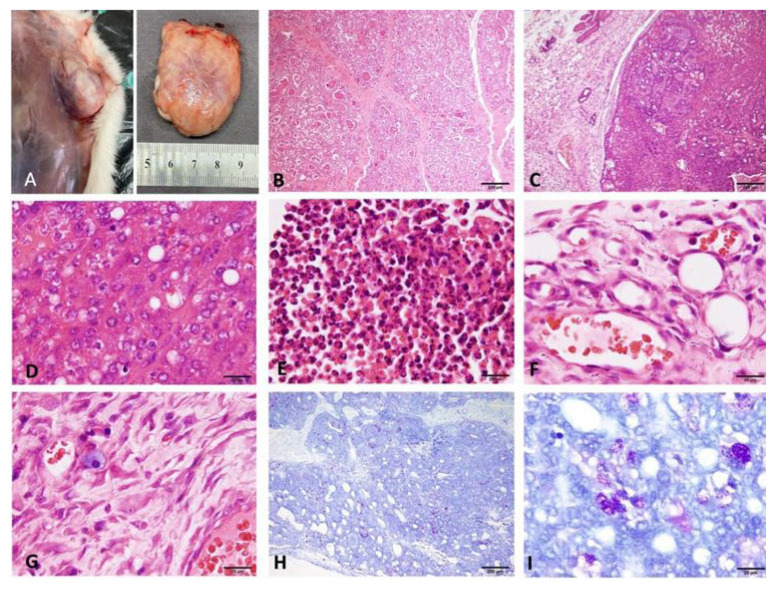
(A) Solid mammary tumor in the right thoracic mammary lobe following DMBA application, (B) histopathological findings of established mammary carcinoma (hematoxylin-eosin), (C) vesicular-type neoplastic cells with high synthesis and proliferation activity, (D) mitotic figure, (E–F) immune cell infiltration in the tumor tissue neutrophils, (G) mast cells close to vessels, (H) invasion of mast cells into tumor tissue (toluidine blue), and (I) degranulation of the mast cells in the intratumoral areas (toluidine blue).

In the experimental group, 100% of the 12 rats administered intragastric DMBA developed mammary tumors over 6–8 months. The tumors exhibited different histopathological subtypes: lipid-rich carcinomas, tubular carcinomas, and solid carcinomas. By using toluidine blue, mast cell invasion was observed in the peritumoral and intratumoral areas of the tumor tissue, regardless of histological subtype. Additionally, a high rate of degranulation (activation) was observed in these mast cells (Figures 1A–1I).

**Figure 2:**
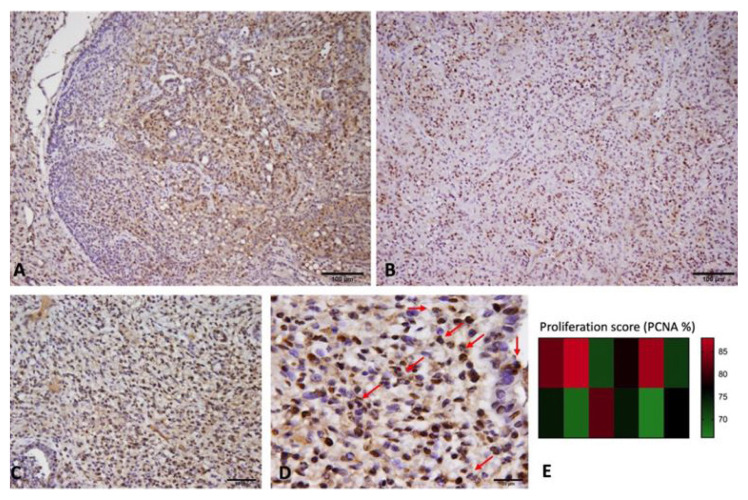
(A–D) Immunohistochemical expression of PCNA in rat mammary tumor tissues; high PCNA-expressing neoplastic cell nuclei and mitotic figures are indicated by the red arrows in (D). (E) Heat map graph of the proliferation index of the experimentally generated mammary tumors.

Microscopic analysis of the mammary tumor tissues using PCNA immunohistochemistry as a proliferative marker revealed a high rate of proliferation of tumor cells in the DMBA-induced mammary tumor compared to the control mammary tissue (Figures 2A–2E).

**Figure 3:**
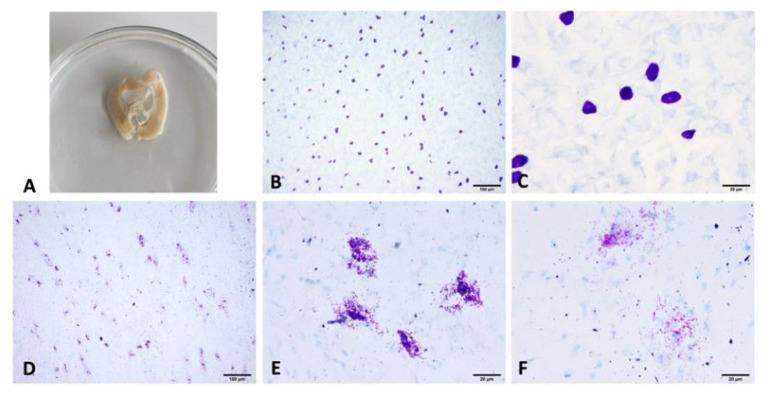
(A) Excised mesenteric tissue with intestines, (B–C) control group rat mesenteric mast cells in granulated form, (D–F) severe degranulation of the mesenteric mast cells of mammary tumor-induced rats, and (E–F) high magnification images of activated mast cells and mast cell granules dispersed in the extracellular environment (toluidine blue).

The mesentery tissue, the area where mast cells are concentrated, was evaluated to analyze the effects of proliferative mammary tumors on systemic mast cells. Mast cells stained purple with toluidine staining of the mesentery tissues were seen in two functional morphologies: granulated (inactive) and degranulated (active) (Figures 3A–3F). The level of degranulation of mast cells in the mesenteric tissue was scored. The microscopic evaluations revealed predominantly granulated morphology or mild degranulation in the control group. In the tumor group, severe degranulation of the mesenteric mast cells was observed.

**Figure 4:**
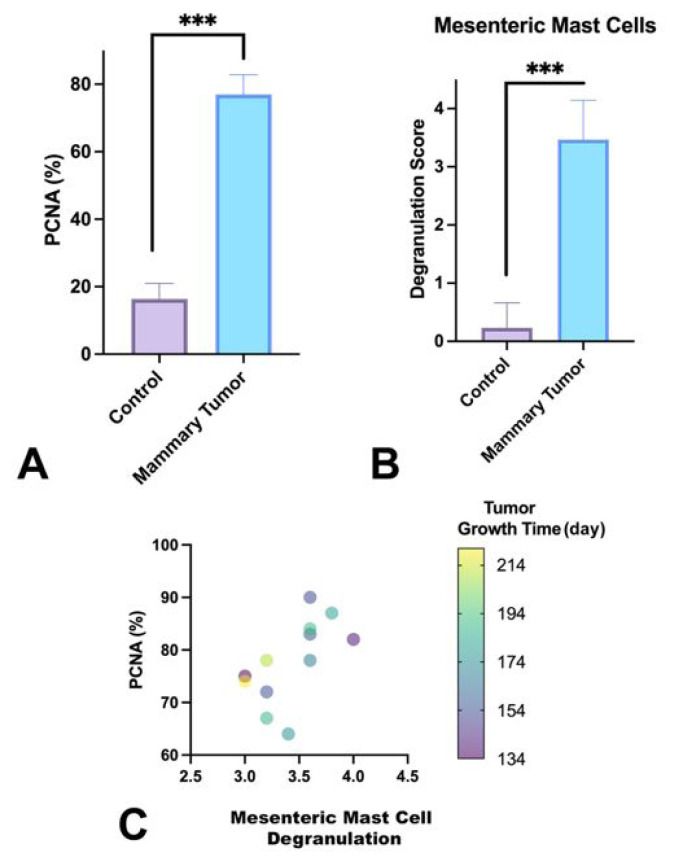
(A) Graph of the PCNA expression values of the control mammary tissues and mammary tumors, (B) graph of mast cell degranulation scores of the control mammary tissues and mammary tumors, and (C) graph of the relationship between PCNA expression value, mesenteric mast cell degranulation, and time to tumor growth.

The PCNA scores for the tumor tissue were significantly different (p < 0.001) than those of the control mammary tissue (Figure 4A). No significant correlation was found between the time to tumor formation and PCNA percentage in the correlation analysis. The severity of mast cell degranulation was compared between both groups, and a statistically significant (p < 0.001) increase was found in the tumor group compared to the control (Figure 4B). Correlation analyses were performed between the degranulation of the mesenteric mast cells and the proliferation score of mammary tumor tissue and the time to 3 cm tumor (Figure 4C). A mildly significant correlation was found between proliferation percentage determined by PCNA immunohistochemistry and mast cell degranulation (p = 0.033 and r = 0.601). There was no correlation between time to tumor formation and mesenteric mast cell degranulation.

## 4. Discussion

Mammary tumors developed in all 12 six-week-old rats designated as the tumor group with a single dose of DMBA. According to the PCNA staining results of the tumors obtained, the proliferation index of the formed tumors was high, and no secondary organ metastasis was observed. These findings are consistent with previously established tumor model studies. In mammary tumor tissues induced by DMBA, the infiltration of mast cells is a normal process of human pathology [20].

In a recent study [21], mammary tumors were experimentally induced chemically in rats, and the increase in the number of mast cells and the Ki-67 proliferation index were evaluated. In that study, tumor proliferation decreased (lower proliferative index) when mast cell degranulation was inhibited before tumor development. In this study, the increased systemic degranulation of mast cells in tumor formation parallels their research.

Another study [22] reported increased mast cells in chemically induced mammary tumor tissues and regional lymph nodes. The number of mast cells increased in the mammary tumors compared to the control mammary tissue, and this finding is similar to that study. However, metastasis was not observed in our research, which could be considered one limitation of this study.

Studies investigating systemic mast cell activation in malignant tumors are relatively low. One study associated systemic MCAS with solid tumors in clinical data and suggested that MCAS may be a risk factor for tumor development. However, all patients with a history of cancer were diagnosed with cancer before MCAS, and the majority of the patients evaluated in the study had breast cancer [11]. In another study, high tryptase levels were detected in the serum of untreated early-stage breast cancer patients [23]. This study’s lack of evaluation of serum tryptase or other mediators is a limitation. However, the presence of systemic mast cell activation was first described in an experimentally induced solid tumor. We believe that we made this identification by demonstrating severe degranulation of mast cells in the mesenteric tissue [24], the site of their predominant concentration. The results suggest that mesenteric mast cell activation develops after tumor development.

Activated mast cells selectively recruit immune cells to tissue sites through multistep mechanisms that may include upregulation of adhesion molecules on vascular endothelium and altered vascular permeability [25–27]. Excessive systemic activity of mast cells can positively or negatively affect the course of cancer. In postmenopausal stage IV cancer, a highly aggressive uterine cancer resistant to two lines of chemotherapy, the agents used for the treatment of MCAS that developed after cancer diagnosis provided the patient with 24–month radiographic and biomarker remission [28]. These data suggest that MCAS may aggravate the course of cancer. In our study, a weak positive correlation was found between the severity of proliferation of mammary tumor cells and systemic mast cell activation. This result may indicate that tumor-induced mesenteric mast cell activation may negatively affect mammary cancer prognosis. However, it may be difficult to administer chemotherapy to a patient with a risk of severe hypersensitivity reactions [29]. Therefore, based on the results of this study, it is necessary to keep in mind the possibility of MCAS for solid carcinomas such as breast cancers in humans. There is currently no well-recognized and long-standing protocol to guide the safe administration of chemotherapy in patients with MCAS [30]. This points to a current treatment protocol that allows for the cautious administration of chemotherapy for patients at high risk of MCAS.

Although it was reported in a case study that MCAS developed secondary to radiation therapy applied to a stage II invasive ductal breast cancer patient [31], it is not yet known at what stage of cancer development MCAS occurs. Considering the impact of inflammation on cancer development and the roles of mast cells in inflammation, an initial diagnosis of systemic mast cell activation may be necessary for detecting and preventing neoplasia. In addition, the known tumorigenic effects of mast cells may be exacerbated by MCAS, characterized by increased mast cell activity, and tumor development may be suppressed by treating MCAS.

As with all cancers, one of the most important factors determining success in breast cancer treatment is the diagnosis of the disease in its early stages, and biomarkers contribute to this. Mast cells and mast cell mediators (e.g., tryptase) may be essential candidate markers for predicting the diagnosis and progression of cancer [23]. They may even be used as targets for new therapeutic approaches. However, before this, a deeper understanding of their biology and a better definition of their roles with their activation and localization are required.

As a result, there have yet to be studies on the development of solid tumors in patients with MCAS. In this study, systemic mastocytosis was observed in rats with cancer, especially in the mesenteric tissue. When the degranulation status of these mast cells is compared to control rat tissue, there is a visible increase in the number of degranulated mast cells. In addition, according to the results of this study, it can be interpreted that the risk of solid tumor formation increases in patients with systemic MCAS. However, more studies are needed to establish this relationship.

## 5. Conclusion

The rat mammary gland consists of six pairs of mammary glands that develop lobuloalveolar structures resembling those of humans [32]. Rat mammary tumor models are known to develop hormone receptor-positive (HR+) and estrogen-dependent mammary tumors with histopathological features similar to human lesions [33]. These features make rats useful models for studying HR+ breast cancer in vivo, with the potential to provide even more benefits. The DMBA-induced model is one of the most widely used models as it generates tumors as in humans. However, it still has several disadvantages in modeling mammary cancer in vivo. The first one is that it cannot accurately model human breast cancer in vivo due to the absence of this carcinogen in the human environment and organism [34]. Second, compared to humans, carcinogen-induced adenocarcinomas in rats tend to be characterized by dominant epithelial components and may exhibit a papillary appearance. Another feature of carcinogen-induced tumors in rats that differs from human breast cancers is the low incidence of metastasis [35]. Despite these limitations, rat models are valuable research tools for studying human malignancies, including HR+ breast cancers.

It has been repeatedly shown that mast cells can promote or inhibit cancer development and growth. To our knowledge, no study has yet been conducted on the development of experimental solid tumors and systemic mast cell activation. This study observed mast cell activation in rats with mammary tumors, particularly in mesenteric tissue. When the degranulation status of these mast cells was compared with control rat tissue, a significant increase in the number of degranulated mast cells was observed. Our results suggest that mesenteric mast cell activation develops following tumor development. However, it is still unclear at which stage of tumor development mesenteric mast cells are activated and what their role is in tumor development and progression. Future studies may be able to clarify this relationship. Mast cells are cells with high heterogeneity, and one of the limitations of this study is the lack of mast cell immunophenotyping. Future studies focusing on the phenotypic characteristics of systemic mast cell activation may clarify this issue.
